# Spinal Complications of Melanoma: A Case of Acute Paraplegia

**DOI:** 10.7759/cureus.71676

**Published:** 2024-10-17

**Authors:** Lyubomir Gaydarski, Danny Kolev, Deyan Popov, Dimitar Metodiev, Georgi P Georgiev, Boycho Landzhov, Asen Hadzhiyanev

**Affiliations:** 1 Department of Anatomy, Histology, and Embryology, Medical University of Sofia, Sofia, BGR; 2 Department of Neurosurgery, University Hospital “Saint Ivan Rilski”, Sofia, BGR; 3 Department of Neuropathological Laboratory, University Hospital “Saint Ivan Rilski”, Sofia, BGR; 4 Department of Clinical Pathology, Nadezhda Women’s Health Hospital, Sofia, BGR; 5 Department of Orthopedics and Traumatology, University Hospital Queen Giovanna, Institute for Emergency Medicine (ISUL), Sofia, BGR

**Keywords:** acute paraplegia, diagnostics, managment, melanoma, thoracic metastasis

## Abstract

Melanoma is an aggressive cancer with a high potential for metastasis, commonly spreading to organs such as the lungs, brain, liver, and bones. Bone metastases, particularly to the spine, are a frequent complication and can result in severe pain, spinal cord compression, and neurological deficits. Prompt diagnosis and treatment are critical, though managing spinal metastases from melanoma poses significant challenges. We present the case of a 41-year-old man with a history of malignant melanoma who developed acute paraplegia following a pathological fracture of the third thoracic vertebra. The patient reported rapidly worsening back pain and loss of motor function in the lower extremities. Magnetic resonance imaging revealed a metastatic lesion in the third thoracic vertebrae, causing spinal cord compression. An emergency open laminectomy with partial tumor resection and vertebroplasty was performed to decompress the spinal cord and stabilize the spine. Postoperative recovery was remarkable, with significant improvement in motor and sensory function within 48 hours. Histopathological and immunohistochemical analysis confirmed the metastatic melanoma diagnosis. This case highlights the challenges of diagnosing and managing acute paraplegia caused by spinal metastases in melanoma patients. Early recognition of symptoms and timely intervention are crucial to improving neurological outcomes.

## Introduction

Melanoma is an aggressive and potentially deadly form of cancer that typically originates in the skin, often beginning as a mole following DNA damage in skin cells caused by ultraviolet radiation from sunlight or tanning beds [1]. The risk of developing melanoma is particularly elevated in older individuals, those with fair skin, a family history of the disease, or compromised immune systems [1]. While melanoma most commonly arises from melanocytes in the skin's outer layer, it can also develop in other melanocyte-containing tissues, such as the genitourinary and uveal tracts. Despite accounting for less than 10% of all skin cancer cases, melanoma is the most lethal, primarily due to its aggressive behavior and high mortality rate [2]. Melanoma is notorious for its ability to metastasize to almost any organ or tissue, including sites rarely affected by other solid tumors [3]. However, certain areas are more likely to be the first to develop distant metastases. The skin, subcutaneous tissue, and lymph nodes are the most common initial sites for distant metastases, occurring in 42% to 59% of patients across various studies. Visceral metastases account for the initial relapse in about 25% of metastatic melanoma cases, with the lungs (18-36%), brain (12-20%), liver (14-20%), and bone (11-17%) being the most frequently affected organs [4]. The incidence of pathologic fractures, a significant concern in musculoskeletal oncology, is on the rise [5]. This increase is largely attributed to improved survival rates among patients undergoing treatment for various malignancies. It is estimated that approximately 5% of patients with metastatic cancer will develop bone metastases, although the prevalence varies depending on the type of cancer [6]. Spinal metastases, a common manifestation of bone metastases, frequently present with back pain, affecting between 80% and 95% of patients [7]. This pain can manifest as localized discomfort, mechanical pain, or radicular pain. In severe cases, metastatic disease may lead to spinal cord compression, a rare but serious complication that requires immediate intervention, occurring in approximately 10 out of every 100,000 patients [8]. Diagnosing bone metastases from melanoma typically relies on identifying characteristic skin lesions, which are often detected early through dermatoscopy [9]. Additional diagnostic tools include blood tests, X-rays, computed tomography (CT), magnetic resonance imaging (MRI), positron emission tomography (PET) scans, biopsies, pathological examinations, and immunohistochemical analyses [10,11]. However, no pathognomonic signs or symptoms are specific to metastatic lesions originating from melanoma [12]. Treatment for bone metastases from melanoma can be either curative or palliative, with several non-surgical options available, including chemotherapy, radiotherapy, biological therapies, and combination treatments [12]. Surgery is often employed to manage bone metastases, primarily to alleviate symptoms and stabilize bones to prevent fractures [13]. The vertebral column, a common site for bone metastases, requires special consideration due to the increased risks of fractures, spinal cord compression, and immobility [12]. Key surgical options include vertebroplasty, balloon kyphoplasty, and en bloc vertebrectomy [12]. The present case highlights the diagnostic and therapeutic challenges encountered in managing patients with acute paraplegia due to metastatic pathological fractures.

## Case presentation

**Figure 1:**
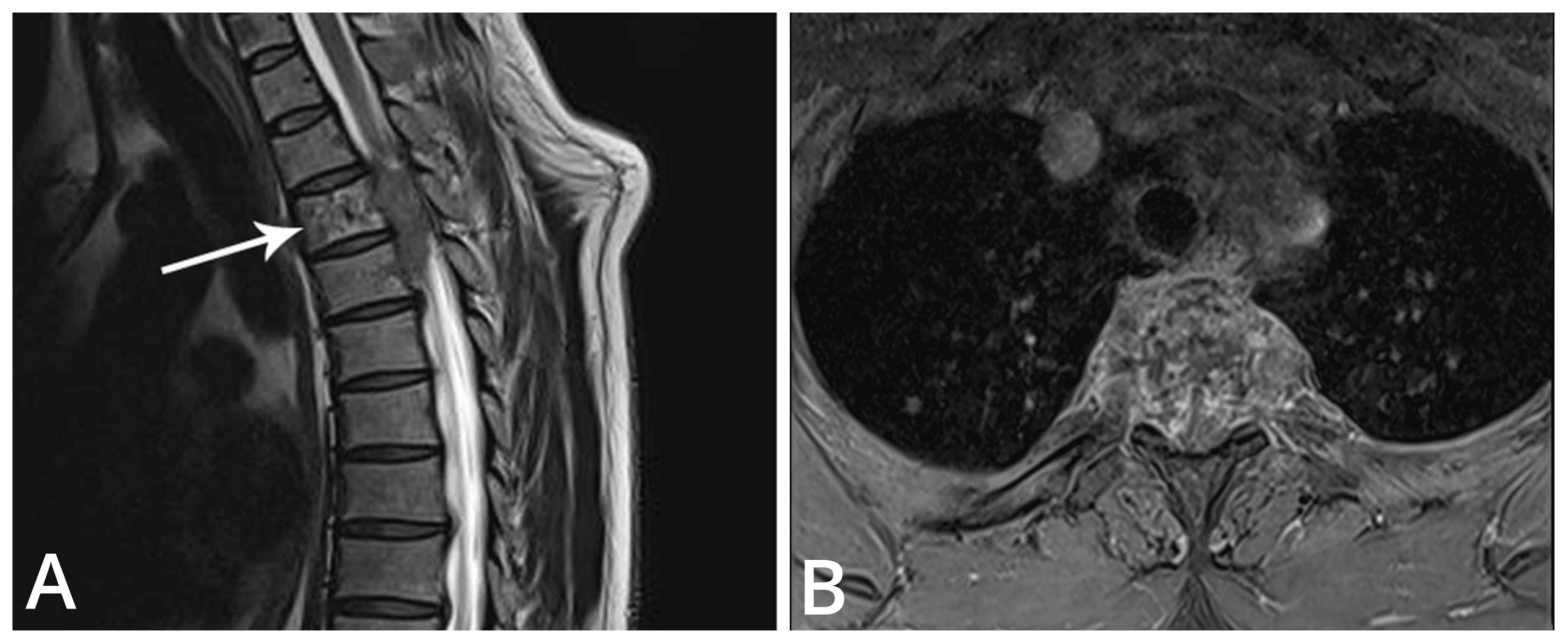
MRI of the patient showing metastatic involvement of the third thoracic vertebra. (A) Sagittal plane; (B) transverse plane. MRI: magnetic resonance imaging

We present the case of a 41-year-old man who arrived with severe thoracic spine pain and a rapidly worsening loss of motor and sensory function in his lower extremities. The patient denied any recent trauma but reported a history of chronic back pain that had sharply intensified the day before admission, ultimately leading to his inability to move his legs upon waking. His medical history was significant for malignant melanoma. On physical examination, he exhibited signs of vertebral syndrome, including complete paralysis (plegia) of the left foot, marked weakness (paresis) in the right foot, and sensory deficits in both lower limbs. Deep proprioception and sphincter tone were preserved, and no additional abnormalities were found. MRI revealed a lesion in the third thoracic vertebra (TH3), showing destruction of the vertebral body with extension into the paravertebral soft tissues and vertebral canal, causing significant spinal cord compression. These findings strongly indicated a pathological fracture of TH3, resulting in severe medullary and radicular compression at the T2-T3 and T3-T4 levels (Figure 1).

**Figure 2:**
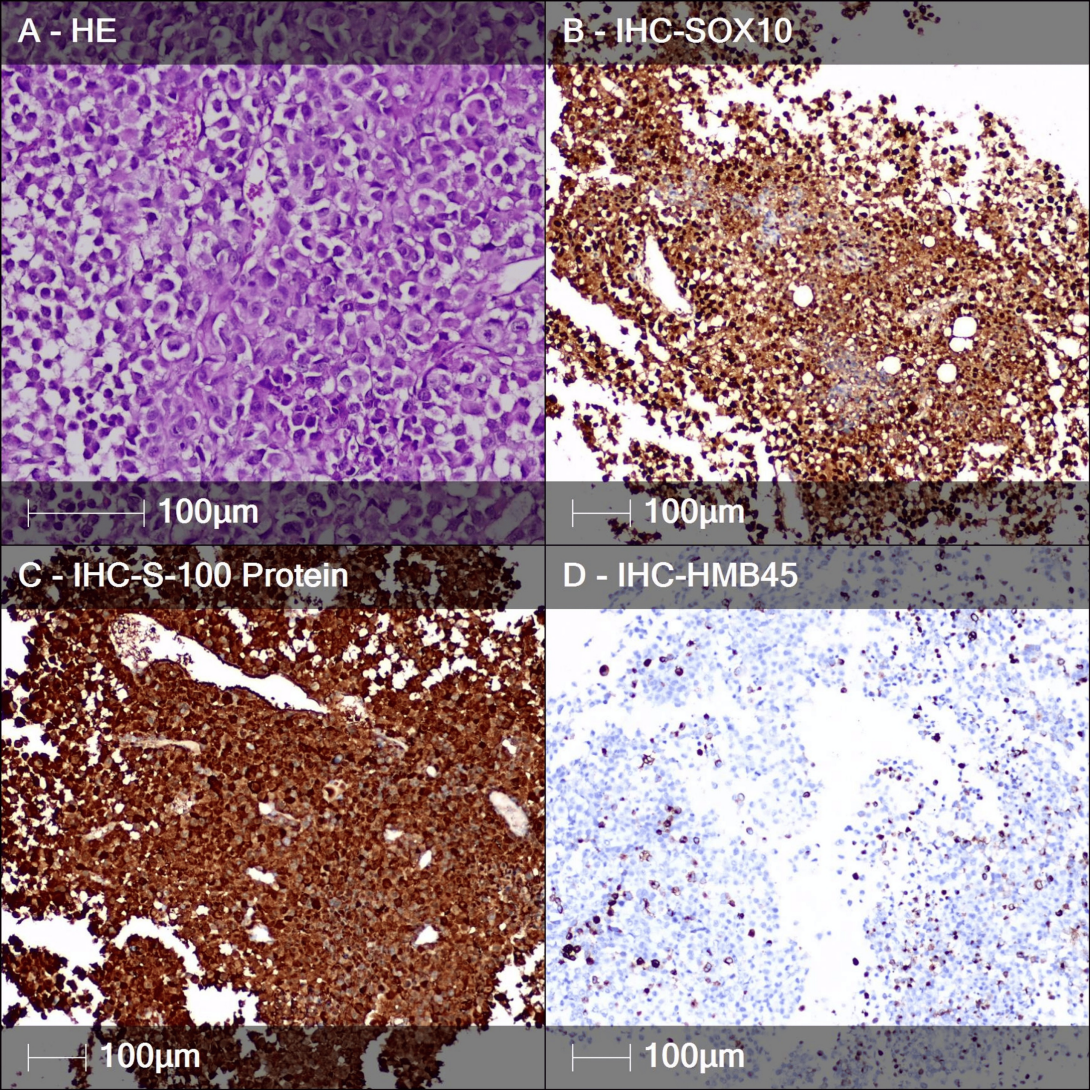
Metastatic malignant melanoma. (A) Large atypical cells with eosinophilic cytoplasm, large round nuclei with vesicular features, and prominent nucleoli (hematoxylin and eosin staining). (B) Positive reaction with SOX10 marker in the nuclei of neoplastic cells (IHC). (C) Positive reaction in the tumor cells with S-100 protein marker (IHC). (D) HMB45 immunopositive in some tumor cells (IHC). IHC: immunohistochemistry

An emergency surgical intervention was performed, including a laminectomy of TH3 with partial tumor resection, bilateral decompression of the spinal cord and nerve roots at the T2-T3 and T3-T4 levels, and vertebroplasty to stabilize the fractured TH3 body. Subsequent histological and immunohistochemical analysis of the resected tissue confirmed the diagnosis of metastatic melanoma. The examination revealed characteristic cellular features of melanoma, including atypical melanocytes with prominent nucleoli, along with a positive staining pattern for melanoma-specific markers (SOX10, S-100, and HMB45), which definitively confirmed the metastatic origin (Figure 2).

Remarkably, within 24 hours post-surgery, the patient regained motor function in his right foot and sensory function in both legs. By 48 hours postoperatively, motor function had returned in both legs. Unfortunately, the patient did not attend any scheduled follow-up appointments, so we cannot provide further information on his condition.

## Discussion

In this paper, we discuss an atypical case of acute paraplegia caused by a pathological fracture due to vertebral metastasis of melanoma. This case underscores the challenges in diagnosing and treating emergency patients with acute neurological deficits from bone metastases. Despite the patient's awareness of his melanoma diagnosis, he overlooked months of dull lumbar pain, leading to a delayed presentation of severe symptoms. Upon his admission, the known history of melanoma directed us toward a diagnosis of a pathological fracture, which MRI promptly confirmed.

Spinal metastases represent a significant concern in oncology, accounting for over 60% of all bone metastases, with breast and prostate cancers being the most frequent sources [13]. About 10-20% of patients with spinal metastases develop spinal cord compression, a critical and often disabling condition that requires urgent intervention [13]. According to a recent study, only 10.6% of all vertebral fractures are pathological, as male patients have a significantly higher risk of pathological fractures [14]. Moreover, according to the same study, the incidence rate of fractures of TH3 is 1.2%, yet the incidence rate of pathological fractures of TH3 is unknown [14]. Diagnosing bone metastases in melanoma typically involves imaging techniques and laboratory tests [12]. Characteristic skin lesions, often detected early through dermatoscopy, guide the initial assessment [9]. Blood tests, particularly for serum calcium and lactate dehydrogenase, can indicate hypercalcemia and serve as biomarkers for disease progression, though they are not always reliable indicators of bone metastases [15]. Imaging techniques such as X-rays, CT scans, MRI, and PET/CT scans are crucial for detecting and characterizing bone lesions [10,11]. X-rays can reveal osteolytic lesions, although their sensitivity is limited, especially when the cortical bone is intact. CT scans offer more accuracy, particularly for detecting axial skeletal metastases, while MRI provides detailed images of soft tissue involvement and the extent of bone infiltration [16]. PET/CT is often considered the gold standard for identifying single bone metastases, although its accuracy is sometimes comparable to MRI [11]. Despite these advancements, no pathognomonic signs are unique to metastatic melanoma lesions, and diagnostic challenges remain, particularly in distinguishing these lesions from those of other tumors [12]. In our case, we opted for an MRI to better visualize the spinal cord. Spinal metastases commonly occur in the vertebral body, particularly in the posterior aspect, influencing treatment strategies [13]. Surgery for spinal metastases is typically palliative, with goals including preserving neurological function, maintaining spinal stability, relieving pain, and facilitating effective radiation therapy. Traditional surgical methods, such as laminectomy, are effective mainly for posterior spinal cord compression and are less suitable for anterior or lateral compressions [17].

Consequently, minimally invasive techniques have become increasingly popular due to their advantages, such as reduced blood loss, less postoperative pain, and shorter recovery times [13]. Advances in intraoperative imaging and navigation have made these techniques more feasible, and polymethylmethacrylate cement has further improved outcomes [18]. Vertebral augmentation procedures, including vertebroplasty and balloon kyphoplasty, effectively manage pain associated with spinal metastases, particularly in cases of vertebral compression fractures [13]. Newer systems provide enhanced control over cement placement, reducing complications. Image-guided tumor ablation techniques also offer a palliative option for patients with painful spinal metastases, especially when re-irradiation is not viable [19]. A multidisciplinary approach to managing spinal metastasis, including surgical, radiological, and other therapeutic modalities, improves patient outcomes [13,17].

In this case, we performed an open laminectomy and tumor resection, followed by vertebroplasty. Additional screw stabilization was not used, as the location of the metastasis and our surgical approach provided sufficient spinal stability after cement application. Our aim was to facilitate a quicker recovery for the patient, who was in stage IV melanoma, and vertebroplasty, being less invasive than metal stabilization, was the preferred option [13,17]. Moreover, the patient’s pulmonary condition restricted the procedure’s duration, and there were institutional constraints related to implant funding. Our primary objective was rapid spinal cord decompression to reverse the patient’s neurological deficits. Postoperatively, the patient showed significant improvement and was discharged without complications. However, the patient did not attend any follow-up appointments, limiting our ability to evaluate the long-term success of the intervention.

## Conclusions

This case underscores the challenges of diagnosing and managing acute paraplegia resulting from pathological fractures due to vertebral metastases of melanoma, a highly aggressive cancer with a propensity to spread to bones. The patient's delayed response to symptoms emphasizes the need for early detection in individuals with known malignancies. Advanced imaging techniques, particularly MRI, played a crucial role in diagnosis, and the combination of open laminectomy, tumor resection, and vertebroplasty effectively stabilized the spine and improved neurological function. However, the lack of follow-up limits our understanding of long-term outcomes. This case highlights the importance of timely intervention, patient education, and adherence to follow-up care in managing metastatic melanoma with spinal involvement.
